# Gamma-aminobutyric acid (GABA) alleviates salt damage in tomato by modulating Na^+^ uptake, the *GAD* gene, amino acid synthesis and reactive oxygen species metabolism

**DOI:** 10.1186/s12870-020-02669-w

**Published:** 2020-10-09

**Authors:** Xiaolei Wu, Qiuying Jia, Shengxin Ji, Binbin Gong, Jingrui Li, Guiyun Lü, Hongbo Gao

**Affiliations:** College of Horticulture, Hebei Agricultural University, Baoding, 071001 China

**Keywords:** Tomato, Gamma-aminobutyric acid, NaCl stress, Na^+^ flux and transportation, *SlGAD* transcriptional expression, Amino acid accumulation, Reactive oxygen species metabolism

## Abstract

**Background:**

Salt stress is a serious abiotic stress that caused crop growth inhibition and yield decline. Previous studies have reported on the the synthesis of gamma-aminobutyric acid (GABA) and its relationship with plant resistance under various abiotic stress. However, the relationship between exogenous GABA alleviating plant salt stress damage and ion flux, amino acid synthesis, and key enzyme expression remains largely unclear. We investigated plant growth, Na^+^ transportation and accumulation, reactive oxygen species (ROS) metabolism and evaluated the effect of GABA on amino acids, especially *SlGADs* gene expression and the endogenous GABA content of tomato (*Solanum lycopersicum* L.) seedlings treated with or without 5 mmol·L^− 1^ GABA under 175 mmol·L^− 1^ NaCl stress.

**Results:**

Exogenous application of GABA significantly reduced the salt damage index and increased plant height, chlorophyll content and the dry and fresh weights of tomato plants exposed to NaCl stress. GABA significantly reduced Na^+^ accumulation in leaves and roots by preventing Na^+^ influx in roots and transportation to leaves. The transcriptional expression of *SlGAD1–3* genes were induced by NaCl stress especially with GABA application. Among them, *SlGAD1* expression was the most sensitive and contributed the most to the increase in glutamate decarboxylase (GAD) activity induced by NaCl and GABA application; Exogenous GABA increased GAD activity and amino acid contents in tomato leaves compared with the levels under NaCl stress alone, especially the levels of endogenous GABA, proline, glutamate and eight other amino acids. These results indicated that *SlGADs* transcriptional expression played an important role in tomato plant resistance to NaCl stress with GABA application by enhancing GAD activity and amino acid contents. GABA significantly alleviated the active oxygen-related injury of leaves under NaCl stress by increasing the activities of antioxidant enzymes and decreasing the contents of active oxygen species and malondialdehyde.

**Conclusion:**

Exogenous GABA had a positive effect on the resistance of tomato seedlings to salt stress, which was closely associated with reducing Na^+^ flux from root to leaves, increasing amino acid content and strengthening antioxidant metabolism. Endogenous GABA content was induced by salt and exogenous GABA at both the transcriptional and metabolic levels.

## Background

In recent years, soil salinization has become an alarmingly severe problem, affecting 10% of the land surface in the world [1] as well as 42.9% of protected soil in China [2]. This issue has become the main obstacle for the sustainable production of protected agriculture. Salt stress harms crops mainly because of the presence of excess ion in soil; among them, Na^+^ and Cl^−^ are not essential mineral but are the main ions causing salt stress injury to plants [3]. Recently, the total soil salt content in greenhouse vegetable fields increased by 69.3% (in which Na^+^ and Cl^−^ increased by 140 and 58% respectively) [4]. Tomato (*Solanum lycopersicum* L.), one of the most widely cultivated vegetable crops, is a moderately salt-sensitive crop [4, 5]. However, soil salinization often severely affects tomato fruit yield and quality by decreasing photosynthetic efficiency and disturbing physiological metabolism due to ion toxicity, osmotic stress, nutrient deficiency, etc. Apparently, compared with the slow progress of breeding [1], the regulation of salt stress tolerance by exogenous substances is a fast and effective method to relieve salt damage in crops, especially by regulating various ion transport pathways and the related metabolism.

Gamma-aminobutyric acid (GABA), a four-carbon non-proteinogenic amino acid, connects the two major metabolic pathways of carbon and nitrogen in plants and its content is significantly higher than that of other non-protein amino acids [3]. It has an important effect on plant growth and abiotic stress resistance as a signal substance or metabolic product by regulating cytoplasmic pH, acting as a temporary nitrogen pool and inducing antioxidant responses [6, 7]. Maintaining cellular ion homeostasis is an important adaptive trait of plant under salt stress [8]. Our previous study showed that exogenous GABA application influenced the absorption and inhibition of mineral elements in cucumber seedlings under NaCl stress and the addition of 5 mmol·L^− 1^ GABA significantly reduced the accumulation of sodium ions in cucumber roots under salt stress [9]. However, there was no strong evidence that GABA directly reduced Na^+^ to relieve salt stress.

Previous studies have demonstrated that the anabolic metabolism of GABA could be activated by salt stress induction and as a result, GABA accumulation has been observed to increase rapidly in a number of plant species, such as tomato, tea, tobacco, and *Arabidopsis* [10–14]. Among these plants, the GABA content was enhanced approximately 20-fold in *Arabidopsis* seedlings under 150 mmol·L^− 1^ NaCl [13], and GABA levels increased significantly in seedlings of lentils treated with 25–100 mmol·L^− 1^ NaCl [15]. Furthermore, it has been demonstrated that the synthesis-related accumulation of endogenous GABA is closely related to exogenous GABA supplementation, which increased by 29% in hulless barley and by 1-fold in *Caragana* treated with 0.5 mmol·L^− 1^ and 10 mmol·L^− 1^ GABA, respectively, under salt stress [16, 17]. Therefore, it is believed that endogenous GABA, which is affected by salt stimulation and exogenous GABA induction, plays a vital role in improving plant resistance to salt stress via regulatory metabolic pathways [18–20]. However, how exogenous GABA affects endogenous GABA synthesis at transcriptional and metabolic levels remains unknown.

The improved salt tolerance due to GABA in plants is related to many physiological metabolic pathways, including the control of reactive oxygen species (ROS) accumulation in tomato [21], the regulation of redox balance and chlorophyll biosynthesis [22], the enabling of cytosolic K^+^ retention and Na^+^ exclusion in *Arabidopsis* [1] and the alteration of cell wall composition [13]. We previously reported that GABA synthesis and supplementation are crucial for enhancing salt tolerance by decreasing ROS generation and photosynthesis in tomato [23] and accelerating NO_3_^−^ reduction and assimilation in pakchoi [24]. Although there are many physiological metabolic pathways involved in the GABA-related plant salt tolerance, the mechanism by which GABA improves plant salt tolerance has not been elucidated clearly. However, these functions of GABA in plants are performed mainly via a short pathway known as the GABA shunt [7]. During this process, GABA accumulation plays a vital role due to irreversible synthesis catalysed by glutamatedecarboxylase (GAD; EC 4.1.l.15) [12, 25] as well as the uptake and transport of exogenous GABA [22].

It has been shown that *GAD* is the most sensitive gene for GABA metabolism in response to abiotic stress. GAD enzyme activity and gene expression levels are closely related to the GABA-mediated enhancement of plant stress resistance [12]. GAD simultaneously catalyses glutamate (Glu) degradation and GABA synthesis. To date, *GAD* genes have been cloned and identified in various plant species, including tomato [25], citrus [26], tea [11] and other plants. Moreover, GABA levels are regulated by *GAD* transcriptional expression and enzyme activity regulation, which contributes approximately 61% to the accumulation of endogenous GABA in NaCl-treated soybean [27] and the GABA content decrease by approximately 50–81% in mature green fruits of *SlGAD2-*suppressed lines [25]. *GAD* gene expression significantly increases GABA accumulation in five wheat cultivars under salt and osmotic stress [28]. *OsGAD2* was the most important gene for GABA accumulation in rice, exhibiting increased activity in vitro and in vivo, showing that transgenic *OsGAD2* had over 40-fold higher activity than wild type (WT) [29]. *SlGAD2* and *SlGAD3* play key roles in regulating GABA levels in tomato fruit, showing that transgenic over-expression lines contained higher levels of GABA (2.7- to 5.2-fold) than the WT [25]. However, there were significant differences in expression sites in different plants under NaCl. *CiGAD1* was expressed in the stem, leaf and seed coat of *Caragana intermedia*, while *CiGAD2* was highly expressed in the bark [17]. The increase in expression of *CiGAD1* and *CiGAD2* induced GABA accumulation within 24 h of salt treatment [17]. *GAD2* expression in all parts of *Arabidopsis* and tobacco was significantly enhanced, accompanied by increased GAD activity and increased GABA content [12, 13]. The *CsGAD* gene enhanced the salt and alkali tolerance of melon by increasing leaf GAD activity and GABA content [22]. However, only a limited number of studies have examined the relationship between *GADs*, GABA and tomato salt tolerance, and the metabolic processes and related metabolism have not been identified.

To experimentally elucidate the relationship between GABA supplementation and tomato plant salt tolerance, we investigated plant growth and changes in Na^+^ flux and accumulation in NaCl-treated with GABA added plants. For the first time, we analysed the transcription level of four *GAD* genes in tomato leaves and detected the amino acid synthesis (including GABA) and metabolism of ROS in leaves to explore the physiological functions of GABA in salt-damaged tomato seedlings. The objective of the study was to elucidate the regulatory mechanism by which exogenous GABA enhances salt tolerance in tomato plants, which might provide new information regarding the molecular regulation of *GAD* genes and the subsequent effect of GABA on ion uptake or metabolic processes in tomato plants under high-NaCl conditions.

## Results

### Effects of exogenous GABA on the phenotype of tomato seedlings under salt stress

**Fig. 1:**
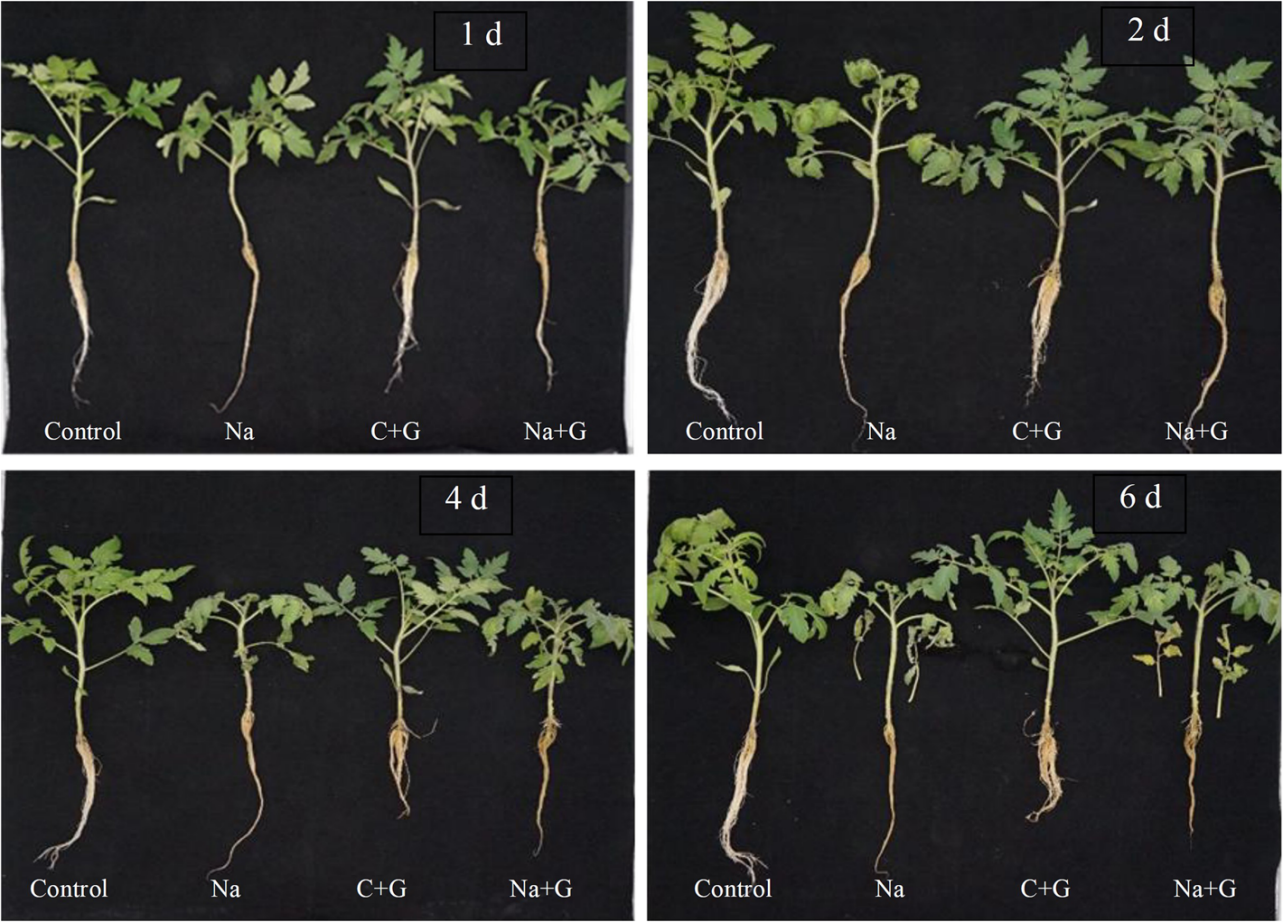
Growth of tomato seedlings in control and NaCl treatment with or without GABA

Extensive damage was apparent in the roots and leaves of seedlings cultivated under salt stress (Fig. 1). The roots of the Na-treatment group exhibited discoloration and atrophy at 1 d after salt stress, and the new leaves wilted at 2 d. The seedling heights were significantly shortened, while the leaves and roots were severely shrunken after salt stress for 4 d. At 6 d of NaCl stress, the seedlings were relatively weak, which manifested as nearly half of the leaves turning yellow and wilting, and the roots lost viability. However, exogenous GABA significantly alleviated the plant phenotypic symptoms of salt injury. At the initial stage of salt stress, the seedlings subjected to GABA treatment did not show root discoloration and their leaves did not wilt, while a small number of aerial roots appeared at 2 d. At 4 d of NaCl and GABA treatment, the seedlings were obviously shorter than those of the control. The leaves remained stretched and did not exhibit obvious wilting and there were more aerial roots than those under NaCl stress. At the end of the experiment (6 d), most of the leaves and roots of seedlings treated with NaCl+GABA remained clearly healthier than the seedlings under salt stress alone.

**Fig. 2:**
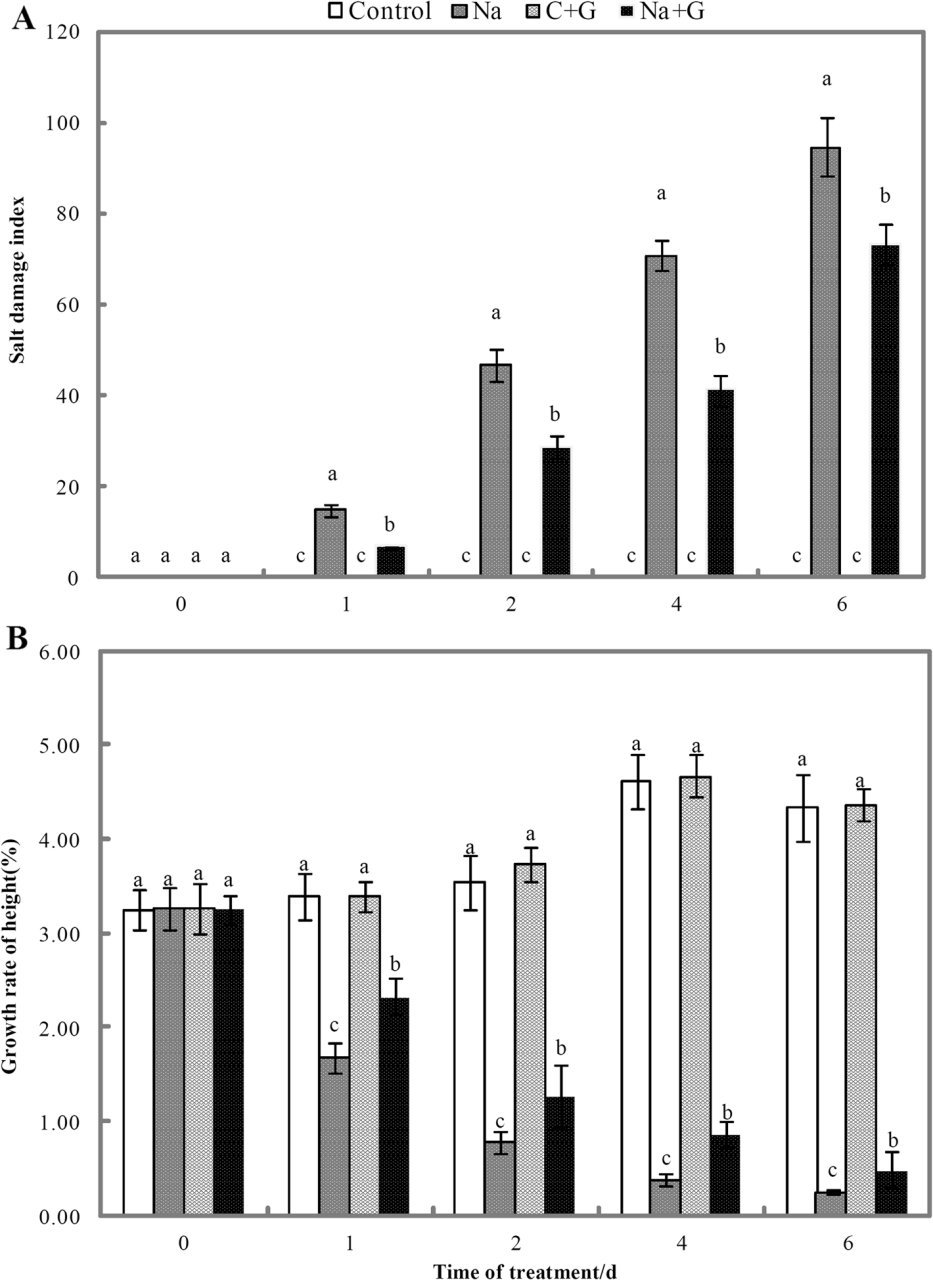
Salt injury index and plant height growth rate of tomato seedlings under NaCl stress with or without GABA. **a**: salt injury index, **b**: plant height growth rate. Note: Each value is the mean ± SD of three independent experiments. Different lower-case letters in each column shape indicate significant difference at *P* < 0.05 by Duncan’s test

The salt damage index of the seedlings treated with NaCl increased with extension of treatment time, but GABA application significantly decreased the salt damage index of the seedlings (Fig. 2a). The salt damage index of the seedlings treated with NaCl+GABA decreased by 54.5, 38.6, 41.5 and 22.5% compared with that of the Na-treatment group. The growth rate in terms of plant height was investigated during the same experimental period (Fig. 2b). The rate of increase in seedling height was approximately 3.2% under normal growth conditions, while NaCl significantly inhibited the height growth of the seedlings; in addition, the rate of increase in seedling height gradually decreased with prolonged treatment time. Although GABA treatment did not enhance plant height under control conditions, GABA alleviate the inhibitory effect of salt stress on seedling height growth under NaCl treatment. The plant height growth rate of the Na + G-treatment group was dramatically higher than that of the Na-treatment group, showing 39.2, 63.6, 126.3 and 92.0% improvement at 1, 2, 4 and 6 d, respectively.

**Fig. 3:**
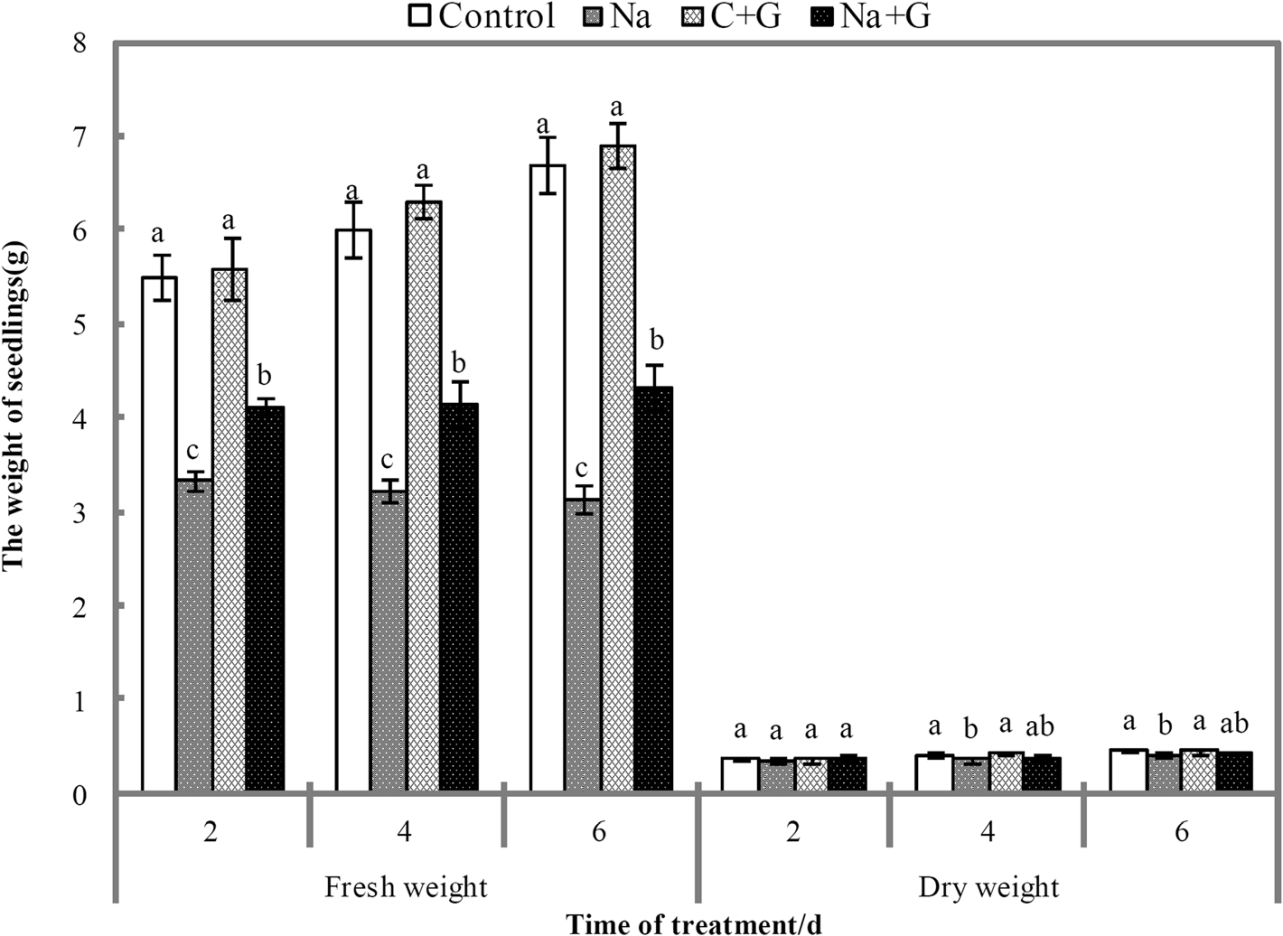
Fresh weight and dry weight of tomato seedlings under NaCl stress with or without GABA. Note: Each value is the mean ± SD of three independent experiments. Different lower-case letters in each column shape indicate significant difference at *P* < 0.05 by Duncan’s test

**Fig. 4:**
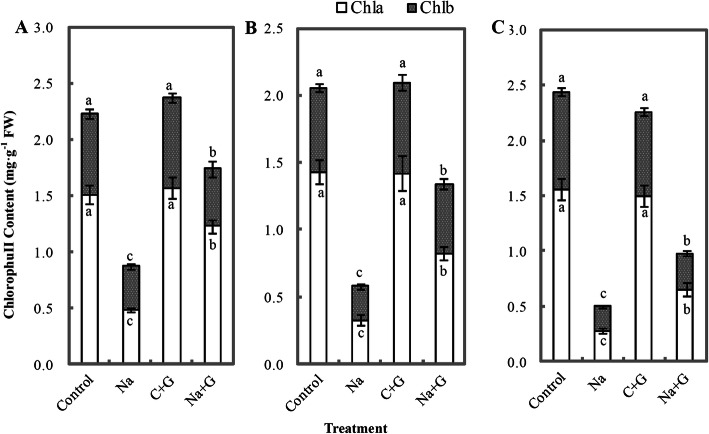
Chlorophyll contents of tomato seedlings under NaCl stress with or without GABA for 2 (**a**), 4 (**b**) and 6 (**c**) d. Note: Each value is the mean ± SD of three independent experiments. Different lower-case letters in each column shape indicate significant difference at *P* < 0.05 by Duncan’s test

The fresh weight of tomato seedlings treated with NaCl and NaCl+GABA was significantly lower than that of C- and G-treatment groups (Fig. 3). Compared with the fresh weight of the NaCl treatment group, that of the NaCl+GABA treatment group was significantly increased by 23.7, 28.8 and 37.9% at 2, 4 and 6 d, respectively, after treatment. The dry weight of the NaCl-treatment group was decreased significantly compared with that of the control treatment group but was not significantly different from that of the Na + G-treatment group. Chlorophyll content was greatly reduced in the leaves of seedlings under NaCl treatment compared with control treatment and decreased gradually with prolonged salt stress (Fig. 4). Exogenous GABA could delay the decrease in chlorophyll a levels under NaCl treatment. During the whole salt stress period, the levels of chlorophyll a and b in the Na + G-treatment group were significantly higher than those in the Na-treatment group, with a range of promotion of 132.1 and 50.0%, respectively, at 6 d after salt stress.

### Effects of exogenous GABA on Na^+^ flux and Na^+^ content in leaves and roots under salt stress

**Fig. 5:**
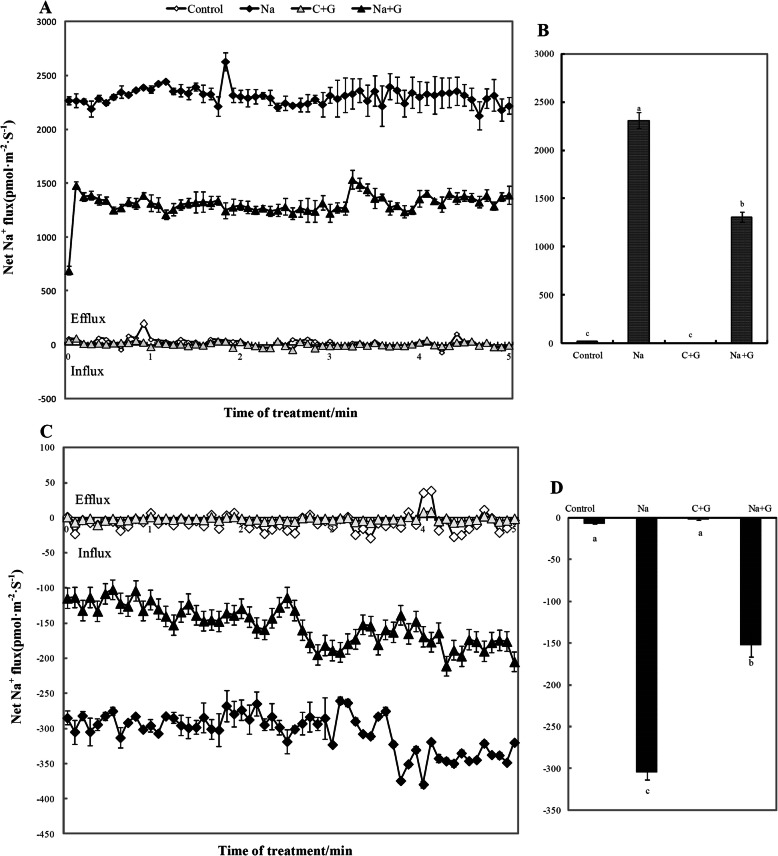
Net Na^+^ flux in leaves and root under NaCl stress with or without GABA 2d after treatment. **a** and **b**: leaves, **c** and **d**: root. Note: The positive value of the ordinate indicates that the ions are discharged (Efflux) and the negative value indicates that the ions are absorbed (Influx). Each value is the mean ± SD of three independent experiments. Different lower-case letters in each column shape indicate significant difference at *P* < 0.05 by Duncan’s test

To further clarify the process of Na^+^ transport from roots to shoots, non-invasive micro-test technology (NMT) was used to measure Na^+^ flux in leaves and roots after salt treatment for 2 d (Fig. 5a and b). Under normal conditions, net Na^+^ efflux in leaves and Na^+^ influx in roots were very low (close to 0) with or without GABA treatment. NaCl stress significantly increased the net Na^+^ efflux in leaves and net Na^+^ influx in roots, with averages of 2309 pmol·cm^− 2^·s^− 1^ and 305.08 pmol·cm^− 2^·s^− 1^ in leaves and roots, respectively. However, the net Na^+^ efflux in leaves and net Na^+^ influx in roots were obviously reduced in GABA-treated seedlings under NaCl treatment, with an approximately 43.2% decline in leaves and a 50.2% decline in roots under NaCl treatment.

**Fig. 6:**
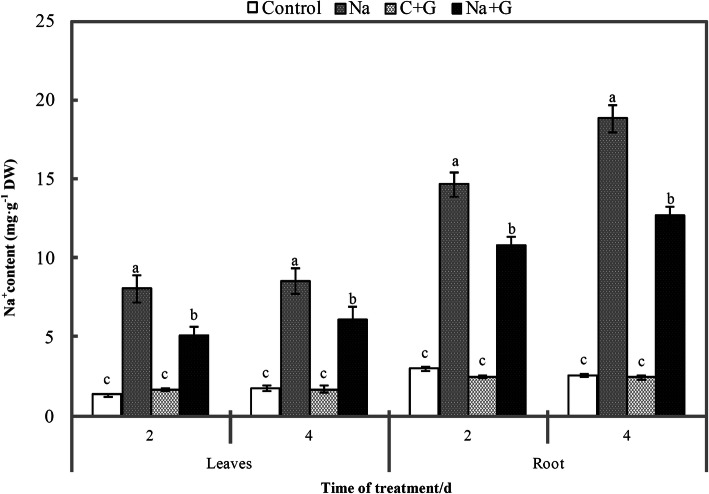
Na^+^ accumulation in leaves and root under NaCl stress with or without GABA 2 d and 4 d after treatment. Note: Each value is the mean ± SD of three independent experiments. Different lower-case letters in each column shape indicate significant difference at *P* < 0.05 by Duncan’s test

Na^+^ accumulation in leaves and roots is the main symptom of salt stress and the results showed that the roots accumulated more Na^+^ than the leaves under all treatments (Fig. 6). There was no significant difference in Na^+^ content in leaves and roots between the control and GABA treatments. The Na^+^ content in the leaves and roots of NaCl-treated seedlings was markedly higher than that of the control. However, exogenous GABA significantly inhibited Na^+^ accumulation in leaves and roots under salt stress, yielding reductions of 28.6 and 32.4%, respectively, relative to control levels at 4 d after treatment.

### Effects of exogenous GABA on the expression levels of four *GAD* genes and GAD activity in leaves under salt stress

**Fig. 7:**
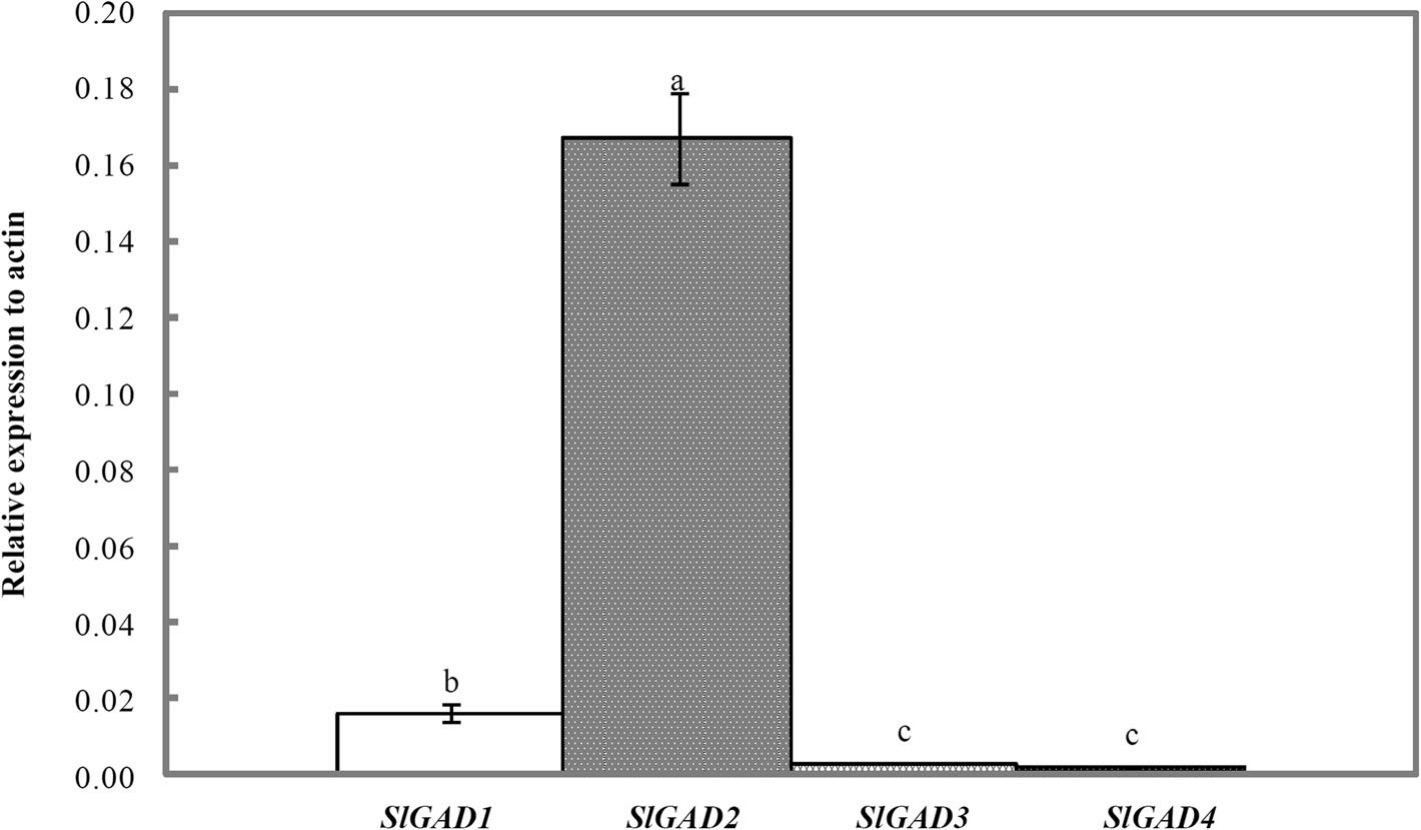
Expression analysis of *GAD* genes in leaves. Plants of 4-week-old were used. Note: Values are means of three biological replicates. Error bars represent the standard error of means; different lower-case letters in each gene indicate significant difference at *P* < 0.05 by Duncan’s test

We cloned the four *GAD* genes, and the conserved region of the sequences showed high homology, with a sequence alignment consistency of 83.73% (Fig. S1). The initial relative expression pattern of all four GAD paralogues in the leaves of tomato seedlings was analysed under normal culture conditions, and the results showed that the four *GAD* gene transcripts exhibited significant expression differences (Fig. 7). Among these genes, *SlGAD2* was the most highly expressed, with approximately 10.7-, 47.8- and 69.6-fold higher expression than *SlGAD1, SlGAD3* and *SlGAD4*, respectively. Among the genes, *SlGAD4* exhibited the lowest expression.

**Fig. 8:**
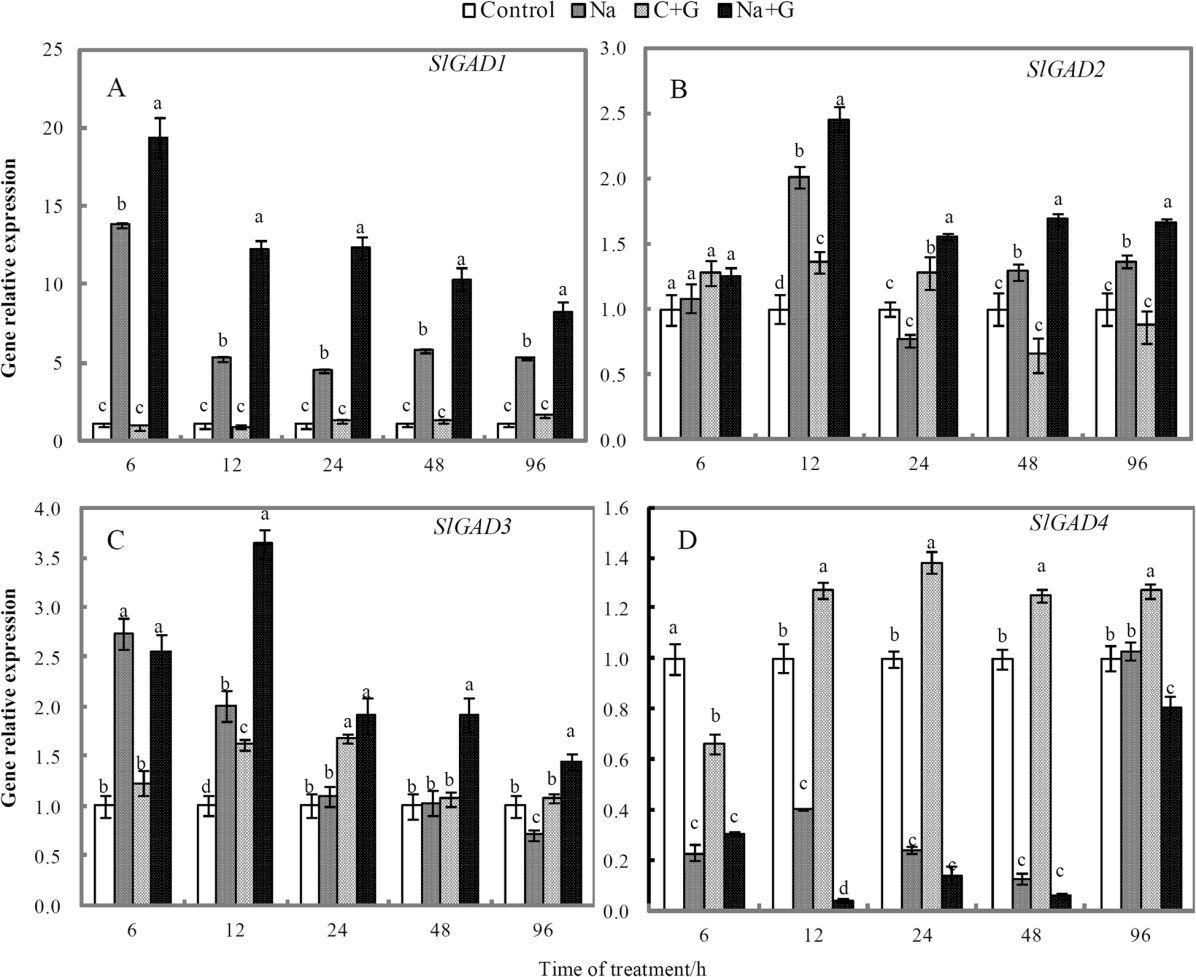
Dynamic changes in the relative expression of four tomato *GAD* genes in the leaves of tomato seedlings subjected to normal culture and NaCl treatment with or without GABA. Note: Gene expression of each treatment of 0 h was taken as 1, and the ordinate was taken as the ratio of gene expression of other time to that of 0 h.The seedlings were shown to one of the following four treatments: Control (white squares), Na (salinity, grey squares), C + G (Control+GABA, grid squares), and Na + G (NaCl+GABA, black squares). Each value is the mean ± SD of three independent experiments. Different lower-case letters in each column shape indicate significant difference at *P* < 0.05 by Duncan’s test

Salt stress significantly increased the expression of *SlGAD1–3* compared with the control (Fig. 8). *SlGAD1–3* showed the same expression trend across the treatments (Na + G > Na > C + G and C). NaCl+GABA treatment induced a greater amount of *SlGAD1–3* expression than NaCl-treatment alone. In contrast, *SlGAD4* expression levels were in the order C + G > Control > Na > Na + G, with the lowest expression level, only 0.039-fold that of the control, observed in the NaCl+GABA treatment at 12 h. Among the four genes, *SlGAD1* exhibited the largest change in transcription level, reaching the highest level after 6 h of Na + G treatment, approximately 19.4-fold that of the control; in the Na-treatment group at 6 h, it had reached 14.5-fold that of the control. The change range of *SlGAD2* and *SlGAD3* was substantially lower than that of *SlGAD1*. At 12 h after Na treatment, the maximum variation in *SlGAD2* and *SlGAD3* was only 2.45-fold and 3.64-fold higher than that of control treatment. *SlGAD4* expression decreased significantly under salt treatment, thus showing the lowest expression among the four genes. However, this expression pattern had little effect on the change of the general trend of the upregulation of *SlGAD* genes.

**Fig. 9:**
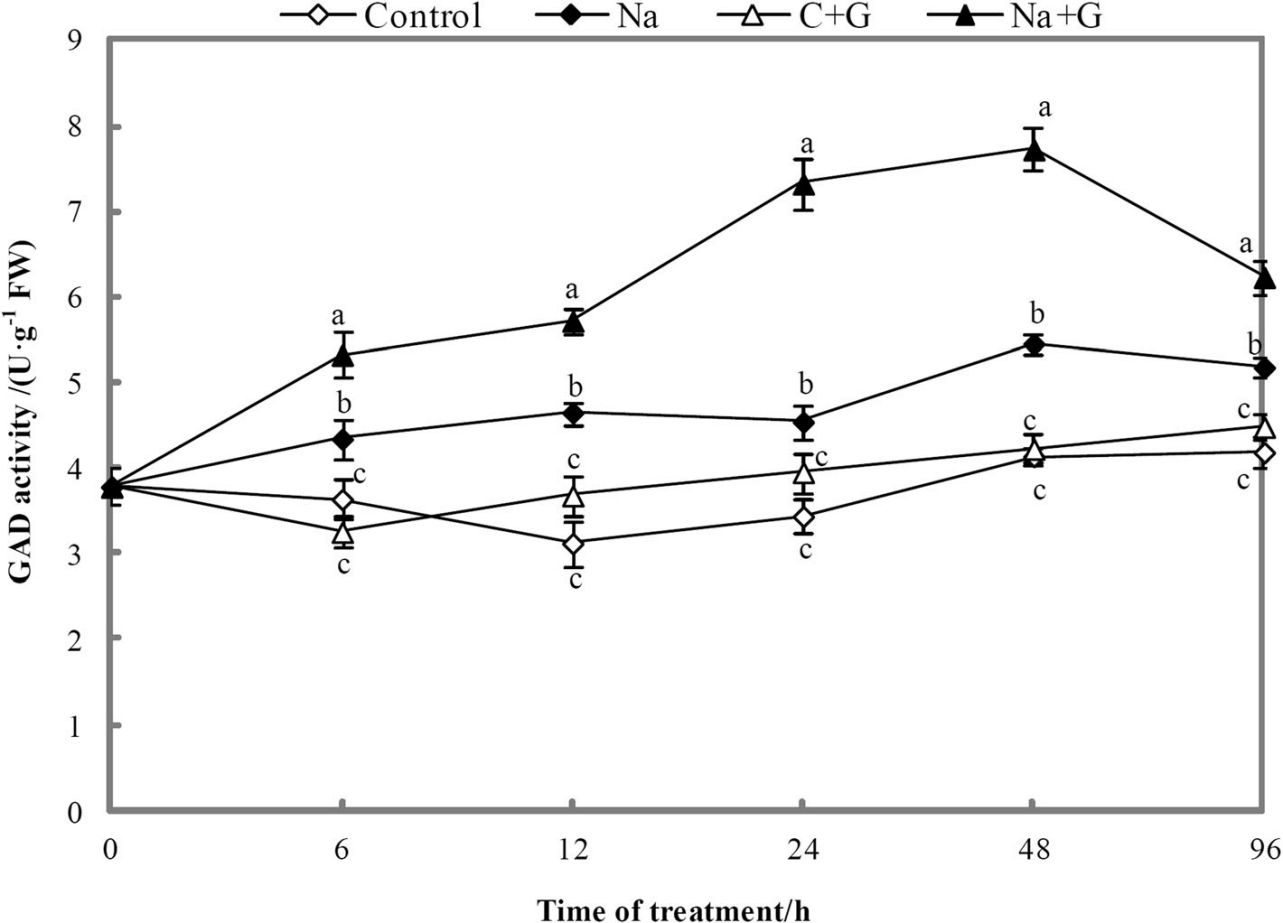
Dynamic changes of GAD activity in the leaves of tomato seedlings subjected to control and NaCl treatment with or without GABA. Note: The seedlings were shown to one of the following four treatments: Control (white diamond), Na (salinity, black diamond), C + G (Control+GABA, white triangle), and Na + G (salinity+GABA, black triangle). Each value is the mean ± SD of three independent experiments. Different lower-case letters in each column shape indicate significant difference at *P* < 0.05 by Duncan’s test

The GAD activity of plants treated with NaCl+GABA or NaCl was significantly higher than that of the control (Fig. 9) and showed an increasing trend for 6–48 h followed by a decreasing trend for 96 h. The GAD activity of the Na+ G-treatment group was the highest during the entire processing period and was markedly higher than that of the Na-treatment group, with an increasing of 20.3 to 61.3%.

### Effects of exogesenous GABA on amino acid content in leaves under salt stress

**Fig. 10:**
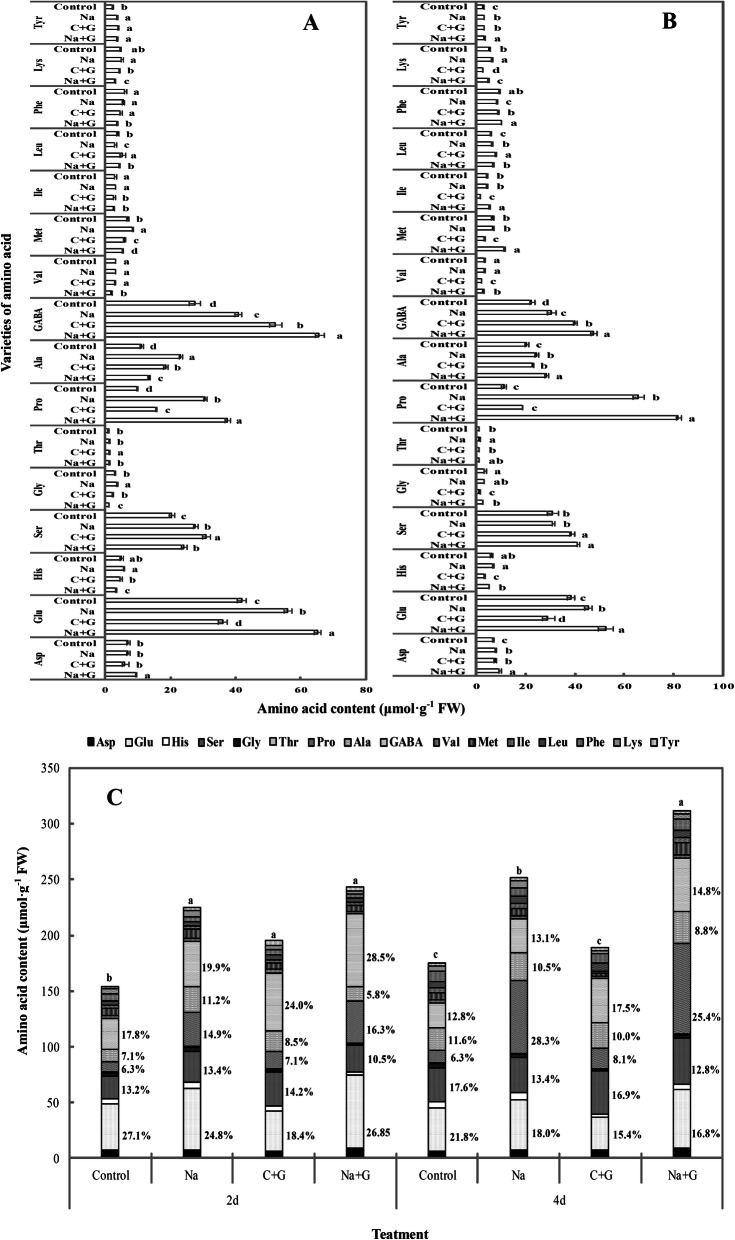
Different amino acids contents in leaves of control and NaCl treatment with or without GABA for 2 and 4 d. **a**:2 d after treatment, **b**:4 d after treatment, **c**:2 and 4 d after treatment. Note: Each value is the mean ± SD of three independent experiments. Different lower-case letters in each column shape indicate significant difference at *P* < 0.05 by Duncan’s test

To analyse the changes in amino acid content, 16 amino acids in leaves from the different treatment groups were detected (Fig. 10a and b). Based on the general trend, the levels of most of the amino acids increased to varying degrees under salt stress compared with the control treatment. Among these amino acids, the levels of methionine (Met), GABA, alanine (Ala), proline (Pro) and glycine (Gly) were significantly higher than those in the control treatment after 2 d of salt stress, and the levels of lysine (Lys), leucine (Leu), GABA, Ala, Pro, threonine (Thr), glutamate (Glu) and aspartic acid (Asp) were significantly higher than those in the control treatment after 4 d of salt stress. GABA, Glu and Pro showed prominent variations among all the amino acids induced by NaCl stress. The levels of GABA, which this article focuses on, were significantly increased by 1.5- and 1.3-fold after 2 d and 4 d of salt stress, respectively. The GABA levels showed the following trend: NaCl+GABA > C + G > NaCl > control, and the levels in the Na + G- and C + G-treatment groups were significantly higher than those after salt stress by 1.6- and 1.3-fold, respectively, 2 d after treatment. Pro levels, which is representative of stress characteristics, significantly increased 1.38-fold under salt stress. The addition of exogenous GABA led to an 18.9% in Pro compared to the level under salt stress (Fig. 10c). However, the addition of exogenous GABA had no significant effect on the Pro level under normal treatment. Glu exhibited a notable increase in the Na-treatment group compared with the control group. Glu levels under salt stress have been further improved by exogenous GABA, with the increases of 16.3 and 15.7%, respectively.

### Effects of exogenous GABA on the activity of antioxidant enzymes under salt stress

**Fig. 11:**
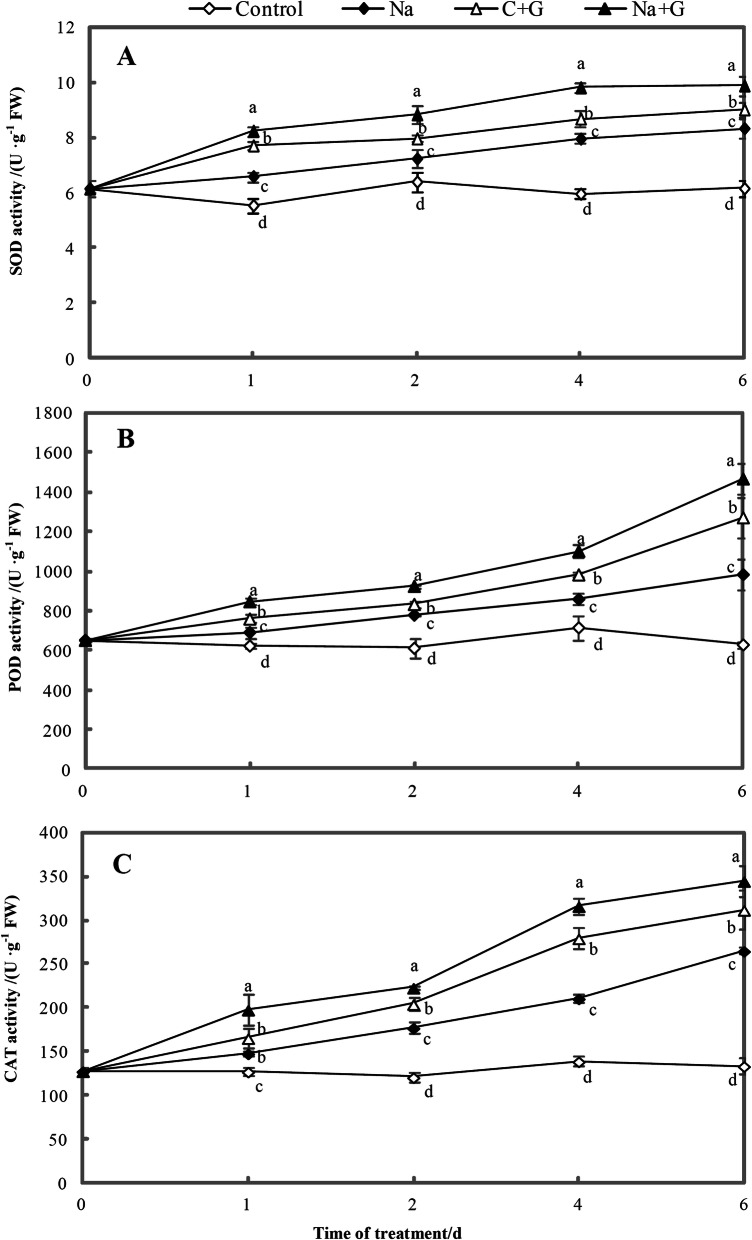
Antioxidant enzymes activities in leaves of tomato seedlings under control and NaCl treatment with or without GABA. **a**: Superoxide dismutase. **b**: Peroxidase. **c**: Catalase. Note: Each value is the mean ± SD of three independent experiments. Different lower-case letters in each column shape indicate significant difference at *P* < 0.05 by Duncan’s test

Superoxide dismutase (SOD) activity in leaves treated with Na, C + G or Na + G gradually increased with increasing treatment time, and all treatment groups showed significantly higher SOD activity than the control (Fig. 11a). The SOD activity in the Na + G-treatment group was the highest and significantly higher than that in the Na-treatment group during the entire treatment process, with increases of 25.1, 22.1, 23.4 and 18.6% at 1, 2, 4 and 6 d, respectively. The NaCl treatment ranked second, with increase of 19.0–35.4% compared with the control.

With increasing treatment time, the peroxidase (POD) activity in leaves treated with NaCl+GABA, C + G or NaCl significantly increased and was obviously higher than that in the control group (Fig. 11b). The Na + G-treatment group showed the highest POD activity, followed by the GABA treatment group and the Na-treatment group, while the Na-treatment group had the lowest POD activity. The POD activity of the NaCl+GABA-treatment group significantly increased by 22.5, 18.7, 28.3 and 49.2% compared with the Na-treatment group and that of the Na-treatment group significantly increased by 11.0–56.5% compared with the control.

During the entire treatment period, the catalase (CAT) activity in tomato leaves showed the following expression trend: Na + G > C + G > Na > C (Fig. 11c). Exogenous GABA treatment significantly increased CAT activity under salt stress compared with that under salt stress alone; the activity was 33.9, 25.9, 50.1 and 30.2% higher than that under NaCl treatment alone.

### Effects of exogenous GABA on reactive oxygen production in leaves under salt stress

**Fig. 12:**
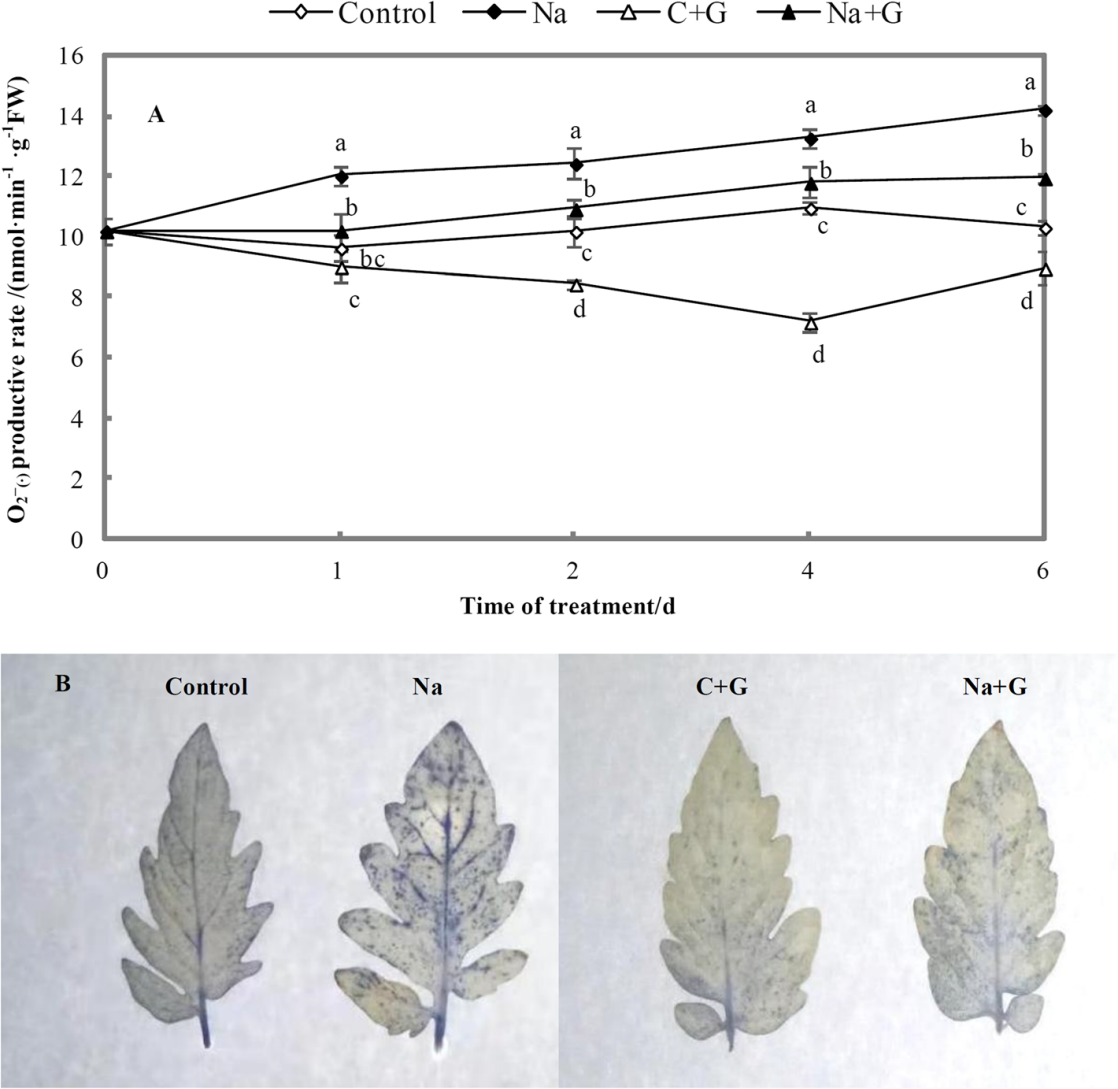
**O**_**2**_^**·-**^ productive rate and tissue staining results in leaves of tomato seedlings under NaCl stress with or without GABA. **a: O**_**2**_^**·-**^ productive rate. **b**: Analysis of ROS production by NBT staining. Note: Each value is the mean ± SD of three independent experiments. Different lower-case letters in each column shape indicate significant difference at *P* < 0.05 by Duncan’s test

Under salt stress, the production rate of superoxide anion (O_2_ $\overline{\bullet}$) in tomato leaves was markedly higher than that in the control group (Fig. 12a). When GABA was added under salt stress, the production rate of O_2_ $\overline{\bullet}$ in leaves was significantly lower than that in the Na-treatment group, with a reduction proportion of closer to 11%. In Fig. 12b, the blue spots indicate the amount of O_2_ $\overline{\bullet}$. The number of blue spots under salt stress was significantly higher than that under the control and GABA treatment conditions,, but the number of blue spots under Na + G treatment was markedly lower than that under NaCl treatment.

**Fig. 13:**
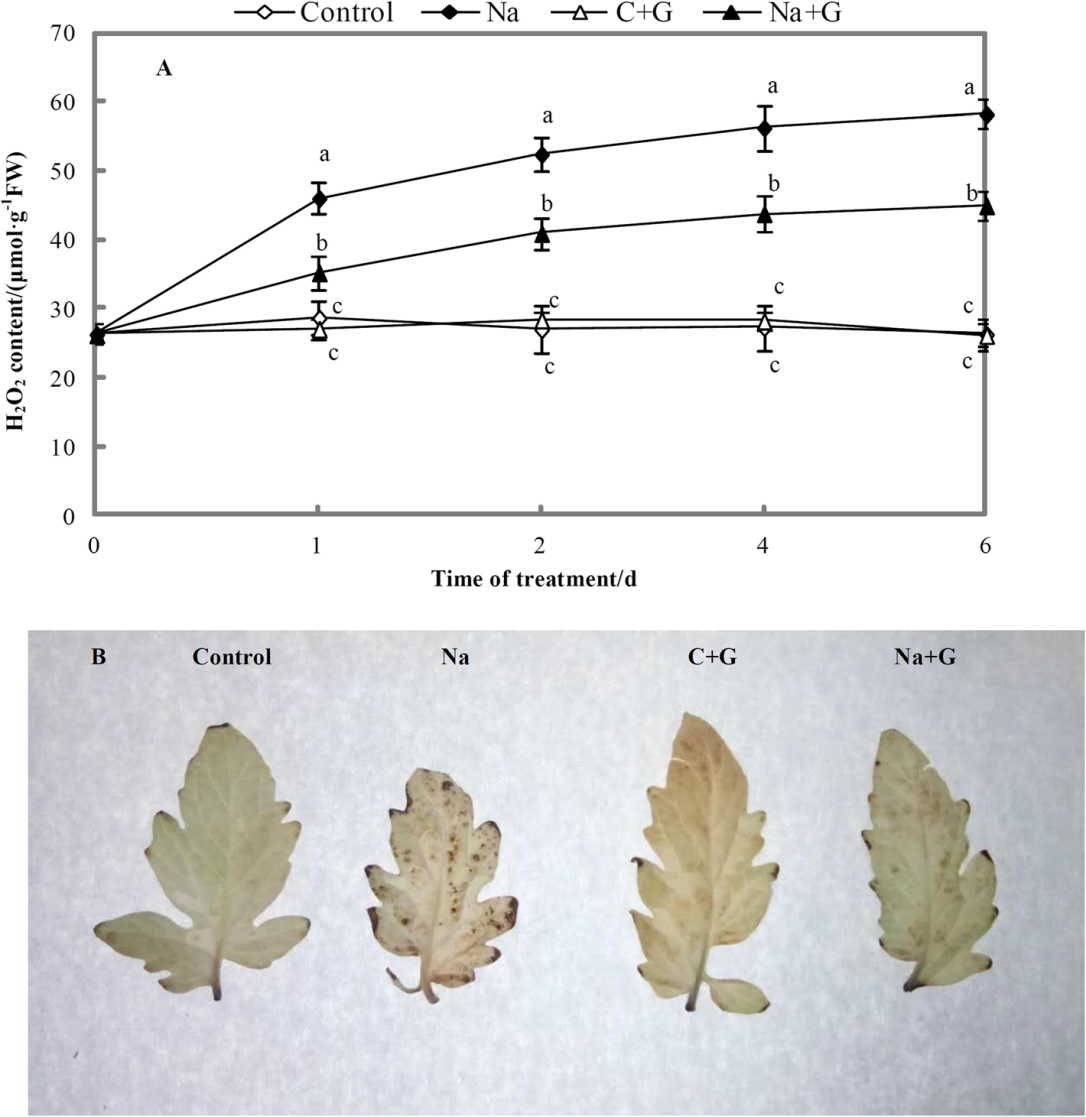
Content of H_2_O_2_ and tissue staining results in leaves of tomato seedlings under NaCl stress with or without GABA. **a**: H_2_O_2_ content. **b**: Analysis of ROS production by DAB staining Note: Each value is the mean ± SD of three independent experiments. Different lower-case letters in each column shape indicate significant difference at *P* < 0.05 by Duncan’s test

Hydrogen peroxide (H_2_O_2_) content was determined using the DAB staining method (Fig. 13a). The H_2_O_2_ content increased with prolongation of salt treatment. The levels of H_2_O_2_ increased significantly under salt treatment, while GABA application significantly inhibited H_2_O_2_ accumulation under NaCl treatment, with reduction of 21.9–23.5%. Under salt stress, the brown spots in tomato leaves indicated the amount of H_2_O_2_ (Fig. 13b). The amount of H_2_O_2_ in leaves under NaCl+GABA treatment was significantly lower than that under NaCl treatment. Exogenous GABA treatment significantly alleviated the active oxygen-related injury of seedlings under salt stress.

**Fig. 14:**
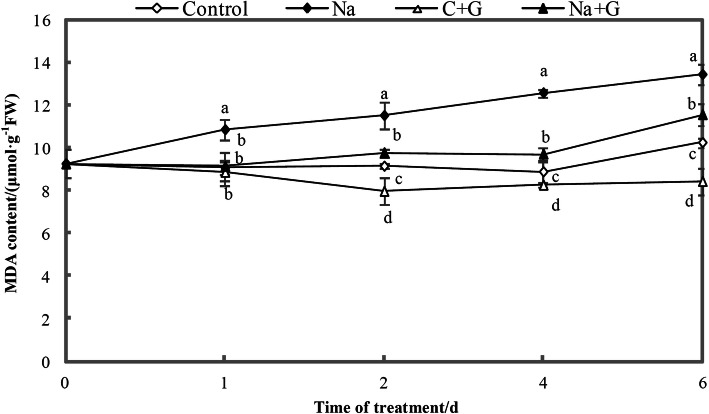
Content of Malondialdehyde in leaves of tomato seedlings under NaCl stress with or without GABA. Note: Each value is the mean ± SD of three independent experiments. Different lower-case letters in each column shape indicate significant difference at *P* < 0.05 by Duncan’s test

As shown in Fig. 14, malondialdehyde (MDA) content in leaves treated with NaCl was markedly higher than that in the control treatment across treatment time. After adding GABA into the nutrient solution of seedlings treated with NaCl, the MDA content decreased significantly, with reductions of 15.8, 15.1, 22.8 and 14.0%. This result indicated that exogenous GABA could significantly reduce the MDA content in leaves under salt stress to alleviate the damage caused by active oxygen in tomato seedlings under salt stress.

### Relationships between phenotypic indexes and levels of reactive oxygen species under salt stress

Correlation analysis of phenotypic and physiological indexes revealed several significant correlations (Table 1). The salt damage index negatively correlated with the rate of increase in plant height, total chlorophyll content and fresh weight and positively correlated with leaf sodium ion content. The sodium ion content significantly correlated with all phenotypic indexes and the levels of all active oxygen species. The levels of ROS significantly correlated with the salt damage index, all phenotypic indicators and sodium ion content.

**Table 1:** Correlation analysis between phenotype and physiological index

Index	Salt damage index	Growth rate of height	Chla+b Content	Fresh weight	Na^+^ content	O_2_^·-^ productive rate	H_2_O_2_ content	MDA content
Salt damage index	1.000	−0.885**	−0.962**	− 0.799**	0.979**	0.828**	0.918**	0.876**
Growth rateof height		1.000	0.913**	0.863**	−0.942**	−0.837**	− 0.920**	−0.820**
Chl(a + b) content			1.000	0.871**	−0.974**	−0.861**	− 0.979**	−0.897**
Fresh weight				1.000	−0.924**	−0.781**	− 0.911**	−0.758**
Na^+^content					1.000	0.824**	0.997**	0.921**
O_2_^·-^productive rate						1.000	0.850**	0915**
H_2_O_2_content							1.000	0.886**
MDA content								1.000

Note:‘*‘shows significant correlation at the level of *p* < 0.05, ‘**‘shows extremely significant correlation at the level of *p* < 0.01

## Discussion

### Exogenous GABA improved salt stress tolerance by alleviating phenotypic symptoms of salt damage

Biomass is a comprehensive indicator of the response to salt stress in many crop species. High salt concentrations reduce biomass, as reflected by reductions in crop growth and yield [30]. Previous studies have indicated that salinity typically reduces plant growth [31] by altering the water potential and inducing nutrient deficiency [32, 33]. The exogenous application of GABA has been shown to alleviate the inhibitory effects of salt stress on plant growth by reducing chlorophyll degradation and maintaining high photosynthetic capacity [20]. For example, the fresh and dry shoot masses of GABA-treated lettuce plants were less negatively affected by saline water than non-GABA-treated plants [23, 34]. In the present study, tomato seedlings gradually exhibited typical symptoms of salt damage, including reductions in growth rate, fresh and dry weights and chlorophyll content. Our results also indicated that treatment with exogenous GABA mitigated the damage due to salt stress by increasing growth rate, fresh and dry weights and chlorophyll level (Figs. 1, 2, 3, 4). These results indicated that reductions in growth could be mitigated by the addition of exogenous GABA, consistent with our previous study of cucumber [9]. GABA may have alleviated the inhibitory effects of salt stress on plant growth by preventing stress-induced damage to chloroplast structure.

### Exogenous GABA regulated Na^+^ flux and Na^+^ content in leaves and roots under salt stress

The damage to plant cells under salt stress and the resulting growth inhibition are mainly caused by the excessive absorption and accumulation of Na^+^ [35]. The ability of a plant to minimize the accumulation of toxic Na^+^ in sensitive shoots is a crucial feature of salinity tolerance [36]. Therefore, the accumulation of sodium ions in tissues is often considered one of the indicators of the degree of salt stress injury. There are few studies on the effect of exogenous GABA on the content of sodium ions in plants and it is not clear whether GABA can directly reduce sodium ion content. However, it has been reported that Arabidopsis mutants with high GABA content have significantly lower root sodium flux than those with low GABA content [1]. Our study showed that Na^+^ flux increased significantly under salt stress, and we also observed for the first time that exogenous GABA significantly decreased Na^+^ efflux in leaves and influx in roots (Fig. 5). These data reveal that exogenous GABA can indeed reduce the absorption of sodium ions, and due to the reduction of absorption, the sodium ions transported to the leaves are significantly lower than that under salt stress, thus showing a low efflux phenomenon. This regulation of sodium ion flux may be the reason why the sodium ion content in leaves and roots of tomato decreased significantly under salt stress with exogenous GABA.

The flux of sodium ions in roots and leaves of salt-sensitive varieties were significantly higher than those of salt-tolerant varieties [36]. The content of sodium ion in leaves of a salt-sensitive Arabidopsis mutant was also significantly higher than that of a salt-tolerant mutant [1]. Therefore, we believe that the significant decrease of sodium ion flux and content by exogenous GABA is the important physiological mechanism of alleviating salt stress. Exogenous GABA can inhibit the absorption of Na^+^ by roots and reduce the total amount of sodium ion transported to leaves (Fig. 6). Previous study indicated that GABA can improve the salt tolerance of plants mainly by changing the ion plasma membrane potential difference and osmoregulation of negative ion transporters, thus affecting ion transport [37]. Combined with our analysis of free amino acid content in leaves represented by Pro, we believe that the absorption of exogenous GABA stimulates the synthesis of endogenous amino acids in leaf cells, resulting in a sharp increase in the content of free amino acids in leaves, thus increasing cell osmoregulation substances and inducing a large amount of sodium ions entering vacuoles. This effect alleviates the toxicity of plant cytoplasmic ions. The photosynthetic system of the leaves was protected, and the tomato seedlings showed better phenotypic relief.

### Exogenous GABA elevated GAD activity by upregulating *SlGAD* expression

Four *GAD* genes in the tomato genome have been reported [21, 38, 39]. Previous studies on the *GAD* genes in tomato mainly focused on the effect of *SlGAD1–3* on the accumulation of GABA during fruit development [25]. Our study is the first to detect the relative expression levels of *SlGADs* in tomato leaves under salt stress (Fig. 8). The transcription level of the *SlGAD1–3* gene increased and that of *SlGAD4* decreased significantly under salt stress. Among these genes, the *SlGAD1* gene showed the most sensitive response to salt stress. Moreover, *SlGAD1* level and GAD activity were significantly positively correlated. Therefore, we speculated that *SlGAD1* plays the most important role among the four *SlGAD* genes in altering GAD activity under salt stress.

Previous studies have shown differences among the *GADs* with regard to their expression induced under stress. In poplar, only two of the six *GAD* genes are upregulated under NaCl stress, with levels significantly higher than those of the other four genes [40]. In the current study, *SlGAD1–3* were upregulated, but *SlGAD4* was downregulated, mainly because different isoforms have different functions under stress. In previous work, exogenous GABA improved *PSGAD2* and *PSGAD4* transcription levels compared with those under single hypoxia treatment [41]. Our results showed that the relative expression of *SlGAD1–3* in the Na + G-treatment group was significantly higher than that in the Na-treatment group. The possible reason is that GABA is absorbed by plant roots and transported to leaf cells. During stress, the accumulation of GABA in the cells induced the expression of *SlGAD*s, and the effect was stronger than that of salt stress alone. Previous reports have shown that GAD activity is stimulated by either cytoplasm acidification or rising intracellular Ca^2+^ levels [20, 42]. The strongly induced expression of *SlGAD*s in the presence of exogenous GABA under salt stress should also be related to the changes in intracellular calcium and hydrogen ions.

GABA accumulation in plant cells is generally believed to occur mainly due to the activation of glutamate decarboxylase [43]. GAD activity in hulled barley and poplar leaves has been found to be significantly higher under salt stress [16, 40]. Furthermore, exogenous GABA treatment has been shown to enhance GAD activity under Ca (NO3)_2_ stress [22]. Our results showed that GAD activity in leaves of plants within the Na-treatment group was significantly increased compared with that in plants leaves in control group and exogenous GABA inspired GAD activity over that observed in plants under NaCl treatment (Fig. 9). The change in GAD activity was consistent with the expression patterns of *SlGAD1–3.* As evidence, exogenous GABA was found to induce an increase in endogenous GABA content under stress conditions with the change of *GAD* gene expression and the increase of GAD enzyme activity measured. These results indicated that the increased endogenous GABA contents in leaves was not only absorbed from exogenous GABA but also derived from GABA anabolism at transcriptional and metabolic levels.

### Exogenous GABA increased the amino acid content in leaves under salt stress

GABA is an important component of the free amino acid pool, which plays an important role in regulating the response of plant cells to stress and enhancing the adaptability of plants to stress [16, 20]. Shelp et al. [19] demonstrated that GABA can alleviate salinity injury and mainly acts as an osmotic substance. Our study found that leaves treated with NaCl exhibited increased accumulation of GABA and Glu (Fig. 10). Exogenous GABA further improved the levels of these two amino acids. Glu can maintain the flow of the GABA shunt and TCA metabolism under stress [44]. The increase in Glu content provides a large amount of raw material for the synthesis of GABA for the synthesis of a large amount of GABA. This process can consume H^+^ and thus reduce the degree of acidification of cells. This finding is consistent with our previous study of hypoxia stress [45].

Under salt stress, the normal nitrogen metabolism in the cells is disturbed and tended to accumulate osmolytes [46, 47]. Free amino acids are considered to be osmoregulation substances that protect plants from salt stress by reducing membrane permeability [8, 48]. Pro is the main nitrogen-containing small molecule, which can increase the cytoplasm concentration to prevent vacuoles from absorbing water from the cytoplasm and protect the metabolic centre of the cytoplasm. Exogenous application of GABA has been shown to increase leaf Pro accumulation under stress conditions [49–51]. Our study is consistent with previous studies and found that Pro content further increased with exogenous GABA application under salt stress. Salt stress induced the fluctuation of Ala [39], serine (Ser) [16] and other amino acids [52] in plants, but their changing patterns are not consistent. Whether these amino acids are directly related to the response to salt stress is not clear. We also found that the levels of 11 amino acids fluctuated dramatically. Among them, the five amino acids accounting for the largest proportion are Glu, Ser, Pro, Ala, and GABA. Previous reports showed that some metabolites involved in plant defence responses were synthesized from amino acid metabolic pathways, playing a key role in plant defence response to stress [53, 54]. Glu is the precursor of Pro and GABA and serves as substrate for converting to α-ketoglutaric acid, which is an important intermediate of carbon and energy metabolism [55]. The main function of increased Glu may be to maintain the metabolic balance under salt stress with exogenous GABA. Ala and Ser precursors originate from the glycolysis pathway and play an important role in chlorophyll synthesis and protein processing [56], even if their content changes are not as large or as sensitive as that of Pro. Therefore, we believe that the accumulation of free amino acids is crucial in enhancing tolerance to salt stress, but the amino acids are not merely osmotic adjustment substances. As an important amino acid to connect carbon and nitrogen metabolism, the significance of exogenous GABA inducing amino acid content is not only maintaining metabolic balance to increase tolerance to salt stress but also increasing osmotic adjustment capacity to resist water loss and neutralize excessive Na^+^ in the vacuoles in leaves. This is also confirmed by our determination of phenotype and sodium ion flux.

### Exogenous GABA improved salinity stress tolerance by regulating the antioxidant system

ROS accumulation in plant cells under stress is considered to cause reductions in photosynthetic system efficiency [57]. Induction of the antioxidant defence system can protect plants from ROS accumulation [58]. Salt stress induces osmotic and oxidative stresses that perturb plant metabolism [16], and lead to membrane damage and the accumulation of lipid peroxides [59]. Our results indicated that salt stress significantly affected the activities of SOD, POD and CAT accompanied by a rapid increase in active oxygen metabolites (Fig. 11), consistent with previous studies [23, 60, 61]. GABA was suggested to act as an inhibitor of MDA formation during lipid peroxidation [62]. Liu et al. found that GABA exhibits ROS scavenging ability and can contribute to alleviating stress [63], although studies are required to determine whether GABA can directly scavenge ROS to relieve stress. In the current study, exogenous GABA enhanced the activities of the three enzymes mentioned above, thereby promoting the conversion of O_2_ $\overline{\bullet}$ into H_2_O_2_ and the subsequent decomposition into water and oxygen. The results also showed that both O_2_ $\overline{\bullet}$ and H_2_O_2_ decreased significantly following exogenous GABA application (Figs. 11 and 12). MDA content is regarded as an important indicator of oxidative damage in plants [64]. We observed a significant decrease in MDA content following exogenous GABA treatment under salt stress (Fig. 14). Some studies have proposed that exogenously applied GABA acts as a signalling molecule in the scavenging of ROS and in modulating antioxidant enzyme activities [13, 65]. According to our test results, the accumulation of amino acid in leaves may help to induce the formation of strong hydrogen-bonded water around protein and protect the natural state of cellular protein polymers, to reduce MDA content. Our results clearly indicated that GABA could strongly protect tomato seedlings from oxidative damage and thus enhance NaCl tolerance.

## Conclusions

Exogenous GABA has a positive effect on mitigating salt stress by modulating Na^+^ uptake, amino acid synthesis and ROS metabolism. This alleviating effect on salt stress injury is mainly due to inducing osmotic regulation and antioxidant reactions by endogenous GABA. Endogenous GABA content was induced by salt and exogenous GABA at both transcriptional and metabolic levels.

## Methods

### Plant materials and cultivation

The experiment was performed in a plastic greenhouse at the Experimental Farm of Hebei Agricultural University in Baoding, Hebei Province, during a spring-summer growing cycle (March–July 2019). The seed material was the tomato cultivar *S. lycopersicum* L. ‘Zhongza9’, a salt-sensitive intermediate-type variety [66] with large fruits (70–80 mm) purchased from China Vegetable Seed Technology Co., Ltd. (Beijing, China), which was approved by the National Crop Variety Approval Committee in 1998(Variety registration No.: Guoshencai 98006). Seedlings were transplanted at the four true-leaf stage into 20-L grey plastic pots (ten plants per pot) without bottom holes and filled with 16 L of Hoagland’s nutrient solution (pH 6.5, EC 2.0–2.2) for hydroponics. An air pump was used to maintain normal ventilation. The treatments described below were performed out after 3 days of pre-culture.

### Treatments and experimental design

1. Control: normal cultivation with Hoagland’s nutrient solution.
2. Salt treatment (Na): 175 mmol·L^− 1^ NaCl was added to the nutrient solution.
3. GABA treatment (C + G): 5 mmol·L^− 1^ GABA was added to the nutrient solution.
4. Salt + GABA treatment (Na + G): 175 mmol·L^− 1^ NaCl and 5 mmol·L^− 1^ GABA were added to the nutrient solution.

All plants used for an experiment were germinated on the same day and kept in the same growth environment. In the pre-test, 175 mmol·L^− 1^ NaCl was the concentration for significant phenotypic difference in the plants screened. Unless otherwise specified, all chemicals were of analytical grade and were obtained from Sigma-Aldrich Co., LLC.(USA). For each treatment, the leaves of the second leaf from the top (four replications) were harvested after transplantation, immediately frozen in liquid nitrogen and stored at − 80 °C until further molecular analysis. Leaves and roots (four replications) were harvested after transplantation and stored at − 20 °C until further biochemical analysis.

### Determination of the salt damage index and plant height growth rate

At 0, 1, 2, 4 and 6 d after treatment, 30 seedlings were selected for the treatment for determination of the statistical salt damage index [67], and the rate of increase in plant height was calculated according to the formula (determined plant height - previously determined plant height) /previously determined plant height× 100%.

### Determination of fresh weight, dry weight and chlorophyll content

At 2, 4 and 6 d after treatment, the fresh weight of the plants was determined. Then, the plants were killed at 105 °C in the oven for 30 min and dried at 80 °C to constant weight, and the dry weight of the plants was measured. The chlorophyll content of the seedlings was extracted with acetone-ethanol (1:1) [68].

### Quantification of Na^+^ flux and Na^+^ content in leaves and roots

Quantification of the activity of the Na^+^ efflux system was performed according to the ‘recovery protocol’ [69]. For this assay, the net Na^+^ flux was measured using NMT (YoungerUSA LLC, Amherst, MA, USA), ASET 2.0 (Sciencewares, Falmouth, MA, USA) and iFluxes 1.0 (YoungerUSA) software [70]. After 2 d of treatment, the leaves and roots of the control and the other three treatment groups were rinsed with distilled water and transferred to the measurement solution containing very little salt (0.1 mmol·L^− 1^ KCl, 0.1 mmol·L^− 1^ CaCl_2_, 0.1 mmol·L^− 1^ MgSO_4_, 0.1 mmol·L^− 1^ NaCl, 0.3 mmol·L^− 1^ MES, pH 6.0). Plant specimens were immobilized in the middle of poly-lysine-coated coverslips (2 × 2 cm) in the measuring chamber. The net flux was measured after 15 min (for leaves) and 30 min (for roots) of equilibration in low-Na^+^ solution. The measurement sites in the leaves were mesophyll cells. The measurement site in the root was 100 μm from the root tip, and vigorous Na^+^ flux was observed in our experiment. The magnitude of the steady-state ion flux was calculated from data recorded over a 300-s period. The glass micropipettes and measuring solutions were prepared as described by Lei et al. (2014) [71].

The Na^+^ content was determined as described in Chen et al. (2010) [72]. After 2 and 4 d of treatment, 100 mg of the leaves and roots of tomato seedlings was dried, and 5 ml of HNO_3_ (65–68%) was added to the microwave digestion system for digestion. The digested sample was diluted to the designated volume with deionized water and filtered with a 0.25-μm pore filtration membrane. The Na^+^ content was determined by inductively coupled plasma mass spectrometry (ICP-MS, PerkinElmer Inc., ELAN DRC-e).

### RNA extraction, cDNA synthesis, cloning of *SlGADs* and qRT-PCR for leaves

Leaf samples (100 mg) from tomato plants were obtained after 0, 6, 12, 24, 48 and 96 h of treatment. RNA extraction was performed as described previously with minor modifications [73]. Total RNA was extracted with the Eastep® Super Total RNA Extraction Kit (Shanghai Biotechnology Co., Ltd., Shanghai). The concentration of RNA was quantified using a NanoDrop (NanoDrop 2000) and the integrity of the RNA was verified on a 1% agarose gel. cDNA was synthesized with the Transcript® One Step gDNA Removal and cDNA Synthesis Supermix Reverse Transcription Kit (TransGen Biotech, Beijing).

The full-length sequences of the *SlGAD1*, *SlGAD2*, *SlGAD3* and *SlGAD4* genes in tomato were published in GenBank as AB359913.1, NM_001246893.1, NM_001246898.2 and XM_004237202.4, respectively. The primers used in this experiment are shown in Table 2. The cDNA of the tomato leaves was used as template for PCR amplification. The reaction system was as follows: cDNA template, 2 μL; forward and reverse primers, 0.5 μL; 2× Flash Hot Start Mastermix (dye), 12.5 μL; ddH_2_O, 9.5 μL. The reaction conditions were as follows: 94 °C for 3 min; 35 cycles of 98 °C for 5 s, annealing for 10 s, 72 °C for 15 s; 72 °C for 10 min. The annealing temperatures for *SlGAD1*, *SlGAD2*, *SlGAD3* and *SlGAD4* were 55 °C, 52 °C, 47 °C and 50 °C, respectively. Agarose gel electrophoresis (1.2%) was used to detect the amplified products. The target fragment was recovered by a DNA Gel Recovery Kit (TIANGEN, Beijing). The amplified product was ligated to the pMD19-T cloning vector (TaKaRa, Beijing) at 16 °C overnight and then transformed into *Escherichia coli* DH5α (TransGen Biotech, Beijing). The cells were plated on LB solid medium coated with ampicillin and the plate was inverted and incubated for 12–16 h at 37 °C. The positive spots were picked and sent to Shanghai Biotechnology Company for sequencing, and finally, the full-length sequence of the gene cDNA was obtained. The conserved regions of the *SlGAD1*, *SlGAD2*, *SlGAD3* and *SlGAD4* genes were compared with DNAMAN software.

**Table 2:** List of primers used for Cloning of *SlGADs*

Gene name	Primer name	Primer sequence (5′-3′)
*SlGAD1*	*SlGAD1*-F	CGCTCCCGCATTTATACC
	*SlGAD1*-R	TACAGGATGGCGATGGAA
*SlGAD2*	*SlGAD2*-F	CTCTTTTGCTTTACTCTTTGAT
	*SlGAD2*-R	GGTCCAATTTTACATTGTAGAT
*SlGAD3*	*SlGAD3*-F	ATGGTTCTCTCAAAAA
	*SlGAD3*-R	CTTCCCTAACAAATAGATGC
*SlGAD4*	*SlGAD4*-F	TTCCTCACTTTACGCCAAA
	*SlGAD4*-R	CCTTCAACAAGTAATCCTTCC

To determine the expression levels of *SlGADs* in tomato, qRT-PCR was performed. The primers were synthesized by Shanghai Biotechnology Company. The reaction mixture contained the following: 2 × Super EvaGreen® qPCR Master Mix, 10 μL (US Everbright® Inc); upstream and downstream primers (10 μmol·L^−1^), 1 μL each; cDNA, 2 μL; water added to a final volume of 20 μL. The reaction procedure was as follows: predenaturation at 95 °C for 2 min, followed by 45 cycles of denaturation at 95 °C for 5 s, annealing at 58 °C for 5 s and elongation at 72 °C for 25 s. The experimental results were analysed by the 2^- △△CT^ method. The relative expression level of each *SlGAD* gene was normalized to the expression level of the *Actin-7* gene (GenBank accession number X58253), which was used as an internal control. The primer sequences used in this experiment are shown in Table 3.

**Table 3:** List of primers used for qRT-PCR of *SlGADs*

Gene name	Primer name	Primer sequence (5′-3′)
*SlGAD1*	*qSlGAD1*-F	GGGGCGGTTCGATATTGTCT
	*qSlGAD1*-R	GCAGCACAGCAATGTGTTCA
*SlGAD2*	*qSlGAD2*-F	TGTGATGAGCCCTGAGAAAG
	*qSlGAD2*-R	ATTGGAGTGTCCCACCCTGT
*SlGAD3*	*qSlGAD3*-F	TGACATCGTCAAGGTCCTCC
	*qSlGAD3*-R	CAAAACTCAGCAATTGCCCT
*SlGAD4*	*qSlGAD4*-F	CTCCACCTTTGCTTCTCGCT
	*qSlGAD4*-R	TCTGGCTCCATCCATGTTGT
*SlACT7*	*qSlACT7*-F	ACCACCACTGCTGAACGG
	*qSlACT7*-R	ACCTCTGGGCAACGGAAC

### Determination of GAD enzyme activity and amino acid content in leaves

Leaf samples (100 mg) of tomato plants were taken after 0, 6, 12, 24, 48 and 96 h of treatment. The GAD activity in tomato leaves was determined by using precolumn derivatization with 2,4-dinitrofluorobenzene (DNFB) and reversed-phase high-performance liquid chromatography (RP-HPLC). One unit of GAD activity was defined as the amount of enzyme required to release 1 mmol·L^− 1^ of GABA every 30 min (40 °C) in every gram of plant tissue [74]. After 2 and 4 d of treatment, the amino acid content was determined by precolumn derivatization with DNFB and RP-HPLC [75].

### Determination of antioxidant enzymes and ROS

SOD activity was determined as described by Giannopolitis et al. (1977) [76]. Inhibition of photochemical reduction of NBT by 50% was used as an activity unit (U). POD activity was determined as described by Zeng et al. (1997) [77]. CAT activity was determined as described by Dhindsa et al. (1982) [78]. The activity unit (U) of the enzyme was defined as 0.1 OD per minute. The production rate of O_2_^−^ was determined as described by Wang Aiguo et al. (1990) [79]; histochemical staining of O_2_ $\overline{\bullet}$ and H_2_O_2_ was performed as described by Christensen et al. (1997) [80]; and the MDA content was determined by using thiobarbituric acid [81]. Each process was repeated three times.

### Statistical analysis

The effects of the various treatments were analysed by the SAS 8.1 statistical program (SAS Institute, Cary, NC) using Fisher’s least significant difference (LSD) test in conjunction with one-way ANOVA, with differences evaluated at a 0.05 level of significance.

## Supplementary information

**Additional file 1 **: **Figure S1.** Alignment of conserved region amino acid sequence of four tomato *GAD* genes. Note: Identical and similar base are shown in black.

## Data Availability

The datasets used and/or analysed during the current study are available from the corresponding author on reasonable request.

## Abbreviations

CAT: Catalase
GABA: Gamma-aminobutyric acid
GAD: Glutamate decarboxylase
H_2_O_2_: Hydrogen peroxide
MDA: Malondialdehyde
O_2_$$ \overline{\bullet} $$: Superoxide anion
POD: Peroxidase
ROS: Reactive oxygen species
SOD: Superoxide dismutase
NMT: Non-invasive micro-test technology
Glu: Glutamate
Pro: Proline
Ser: Serine
Ala: Alanine
Met: Methionine
Gly: Glycine
Lys: Lysine
Leu: Leucine
Thr: Threonine
Asp: Aspartic acid
WT: Wild type
