# Supplementary figures and images for: Gamma-aminobutyric acid (GABA) alleviates salt damage in tomato by modulating Na+ uptake, the GAD gene, amino acid synthesis and reactive oxygen species metabolism
Source: BMC Plant Biol. 2020 Oct 9;20:465. doi: 10.1186/s12870-020-02669-w (PMC7547442; doi:10.1186/s12870-020-02669-w)

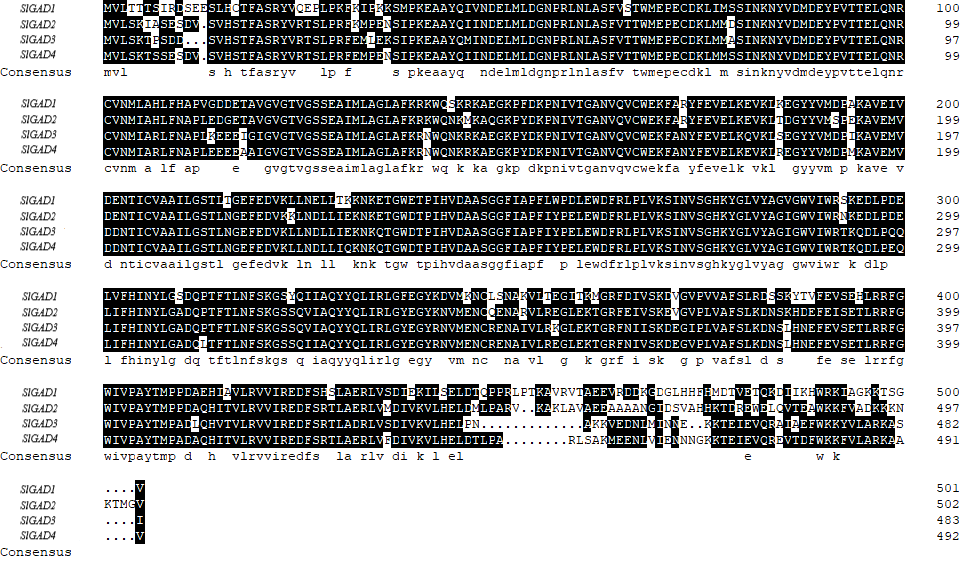

Supplement: Supplementary file 1 — Additional file 1 : Figure S1. Alignment of conserved region amino acid sequence of four tomato GAD genes. Note: Identical and similar base are shown in black. [file 12870_2020_2669_MOESM1_ESM.tiff]
